# A novel method for evaluating combined component anteversion in total hip arthroplasty on cross-table lateral hip radiographs

**DOI:** 10.1007/s00402-023-04825-x

**Published:** 2023-03-11

**Authors:** Dimitris Dimitriou, Christoph Zindel, Elin Winkler, Frédéric Cornaz, Peter Mazel, Patrick O. Zingg

**Affiliations:** Department of Orthopedics, Balgrist University Hospital, University of Zürich, Forchstrasse 340, 8008 Zurich, Switzerland

**Keywords:** Total hip arthroplasty, Femoral anteversion, Acetabular anteversion, Combined anteversion, THA dislocation

## Abstract

**Introduction:**

Accurate measurement of combined component anteversion (CA) is important in evaluating the radiographic outcomes following total hip arthroplasty (THA). The aim of the present study was to evaluate the accuracy and reliability of a novel radiographic method in estimating CA in THA.

**Materials and methods:**

The radiographs and computer tomography of patients who underwent a primary THA were retrospectively reviewed, to measure the radiographic CA (*CAr*), defined as the angle between a line connecting the center of the femoral head to the most anterior rim of the acetabular cup and a line connecting the center of the femoral head to the base of the femoral head to allow a comparison with the CA measured on the CT (*CACT*). Subsequently, a computational simulation was performed to evaluate the effect of cup anteversion, inclination, stem anteversion, and leg rotation on the *CAr* and develop a formula that would correct the *CAr* according to the acetabular cup inclination based on the best-fit equation.

**Results:**

In the retrospective analysis of 154 THA, the average *CAr_cor,* and *CACT* were 53 ± 11° and 54 ± 11° (*p* > 0.05), respectively. A strong correlation was found between *CAr* and *CACT* (*r = *0.96, *p* < 0.001), with an average bias of − 0.5° between *CAr_cor* and *CACT*. In the computational simulation, the *CAr* was strongly affected by the cup anteversion, inclination, stem anteversion, and leg rotation. The formula to convert the *CAr* to *CA_cor* was: *CA-cor = *1.3*Car − (17* In (*Cup Inclination*) − 31.

**Conclusion:**

The combined anteversion measurement of THA components on the lateral hip radiograph is accurate and reliable, implying that it could be routinely used postoperatively but also in patients with persistent complaints following a THA.

**Level of evidence:**

Cross-sectional study, Level III.

## Introduction

Accurate measurement of component orientation is important in evaluating the radiographic outcomes following total hip arthroplasty (THA), as component malposition might be associated with early implant failure, impingement, dislocation, and early implant failure [5, 11, 16, 18, 20, 22, 24]. The concept of combined anteversion (CA), introduced by Ranawat [21] as the sum of acetabular and femoral component anteversion, is gaining popularity as its importance in preventing THA dislocation has been widely demonstrated [7, 10, 17, 30]. Several methods have been proposed for accurate measurement of acetabular cup anteversion on plain radiographs [1, 6, 8, 12, 14, 26]; however, no method exists to assess femoral stem anteversion and consequently CA. Although computed tomography (CT) is considered the gold standard in evaluating CA, plain radiographs are commonly used postoperatively, as CT scans are expensive and expose patients to a considerable amount of radiation [2, 3, 7].

**Fig. 1:**
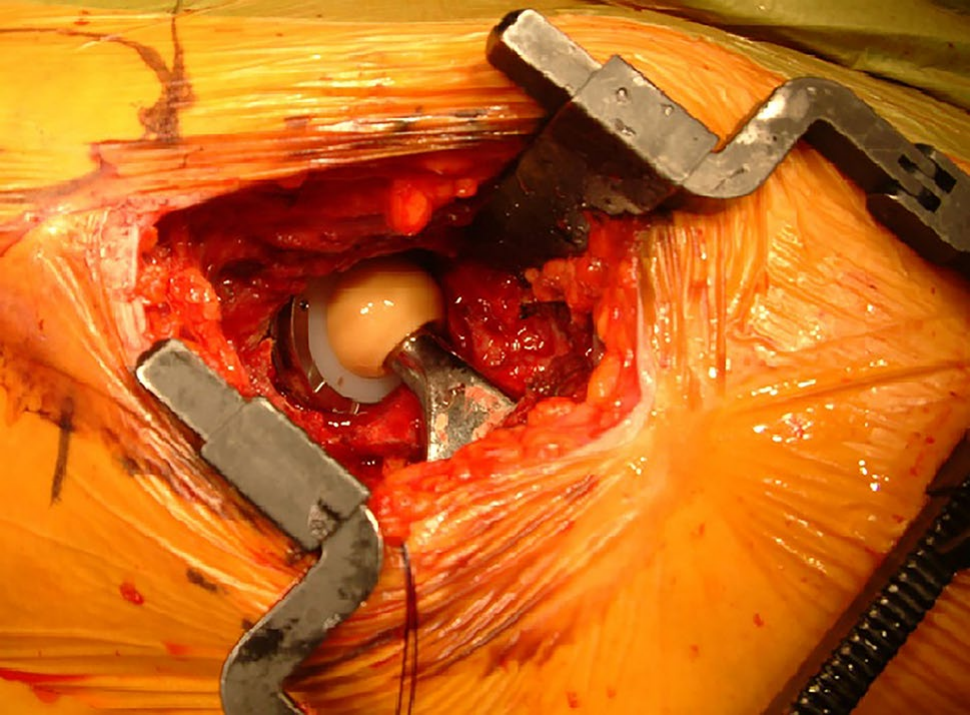
Intra-operative photograph of a right total hip arthroplasty in 15° of internal rotation and 0° of hip and knee extension demonstrating a large proportion of the femoral head being uncovered from the acetabular cup, implying a high combined anteversion of the components

The Ranawat sign [15] was introduced by Lucas and Scott as an intraoperative maneuver to assess CA. Hereby, the operated leg is placed in zero degrees of extension and 45° of internal rotation. If the base of the femoral head is parallel to the acetabular cup, a combined anteversion of 45° could be assumed. Based on the Ranawat sign and the intraoperative observation that the proportion of the anterior femoral head, which was not covered by the acetabular cup, could give an estimation of the CA (Fig. 1), a simple method was developed to assess the CA on cross-table lateral hip radiographs. The aim of the present study was: (1) to verify the novel method with a computational simulation and (2) to evaluate its accuracy and reliability in estimating CA in THA.

## Materials and methods

### Retrospective study design, inclusion, and exclusion criteria

The present study was approved by the state ethical committee (BASEC Nr.: 2022-01344) and was entirely conducted at the authors' institution. The medical records and radiographs of patients who underwent a rotational profile CT scan (including the pelvis and knee) at our institution either as part of a randomized control trial comparing a three-dimensional CT-based preoperative to the standard two-dimensional planning (NCT05120063) or because of hip instability (consecutive patients from 01/2012 until 12/2021), were retrospectively reviewed. The inclusion criteria were adult patients, who underwent a primary THA, and had a CT scan and cross-table lateral hip radiograph, 3 months postoperatively. The exclusion criteria were revision THA, gross hip deformity making complex hip reconstruction necessary, such as greater trochanter advancement, acetabular augmentation, femoral osteotomy, and use of a revision stem, as those patients might have increased risk for THA dislocation, which could have affected the subgroup analysis.

### Patient characteristics, surgical technique, and implants

A total of 154 THA in 148 patients (males: 53, females: 95) with an average age of 59 ± 11 (range: 24 to 91) years were included. The majority of the patients (THA = 108/154, 70%) underwent a rotational CT as part of a randomized control trial, whereas 46/154 (30%) received a CT scan due to an early hip dislocation (< 3 months postoperative). All cases were performed with a standardized minimally invasive direct anterior approach in a supine position using the AMIS^®^ Mobile Leg Positioner [Medacta International SA, Castel San Pietro, Switzerland] under spinal or general anesthesia. An imaging intensifier was used intraoperatively to assess the acetabular cup anteversion in all cases. The implants used in the current study included a cementless press-fit acetabular cup (April [Symbios, Yverdon-Les-Bains, Switzerland] (*n = *108) or Versafit [Medacta International SA, Castel San Pietro, Switzerland] (*n = *46) and a cementless straight femoral stem (Harmony^®^ [Symbios, Yverdon-Les-Bains, Switzerland] (*n = *62) or Quadra-H [Medacta International SA, Castel San Pietro, Switzerland] (*n = *46) and a three-dimensional anatomical stem (SPS^®^ Evolution (*n = *46) [Symbios, Yverdon-Les-Bains, Switzerland].

### Radiographic measurements

A standardized cross-table lateral hip radiograph was performed 3 months postoperatively. This was taken in a supine position, with the foot rotated internally at 15° and the contralateral hip flexed to 90° to prevent interference in the radiographic projection. The cassette was held perpendicular to the examination table and the direction of the beam was parallel to the examination table and 45° cephalad to the long axis of the body.

**Fig. 2:**
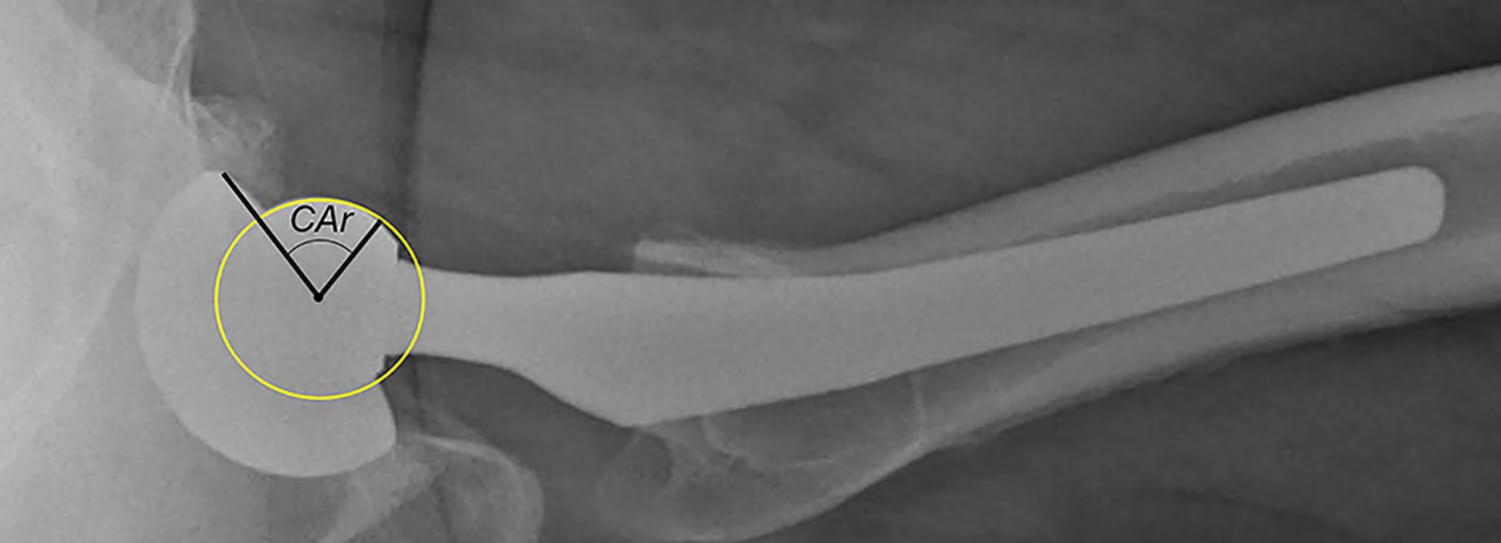
Cross-table lateral radiograph of a left hip demonstrating the method used to measure the radiographic combined anteversion of the components (*CAr*). A best-fit circle was applied to the femoral head. *CAr* was defined as the angle between the line connecting the center of the femoral head (best-fitted circle) to the most anterior rim of the acetabular cup and the line connecting the center of the femoral head to the base of the femoral head, defined as the point where the head loses its sphericity

For the measurement of the radiographic* CA (CAr)* in the cross-table lateral radiograph, the best-fit circle was applied to the femoral head. *CAr* was defined as the angle between the line connecting the center of the femoral head to the most anterior rim of the acetabular cup and the line connecting the center of the femoral head to the base of the femoral head, defined as the point where the head loses its sphericity (Fig. 2).

### CT measurements

CT scans of the pelvis and the distal femur were obtained with a 64-slice CT scanner (SOMATOM Definition AS, Siemens Healthineers) using a standardized protocol. CT images were acquired with automated tube voltage selection (CARE kV, reference 120 kV) and tube current modulation (CARE Dose4D, reference 147mAs), a collimation width of 0.6 mm, a rotation time of 0.5 s, and a pitch of 0.8.

**Fig. 3:**
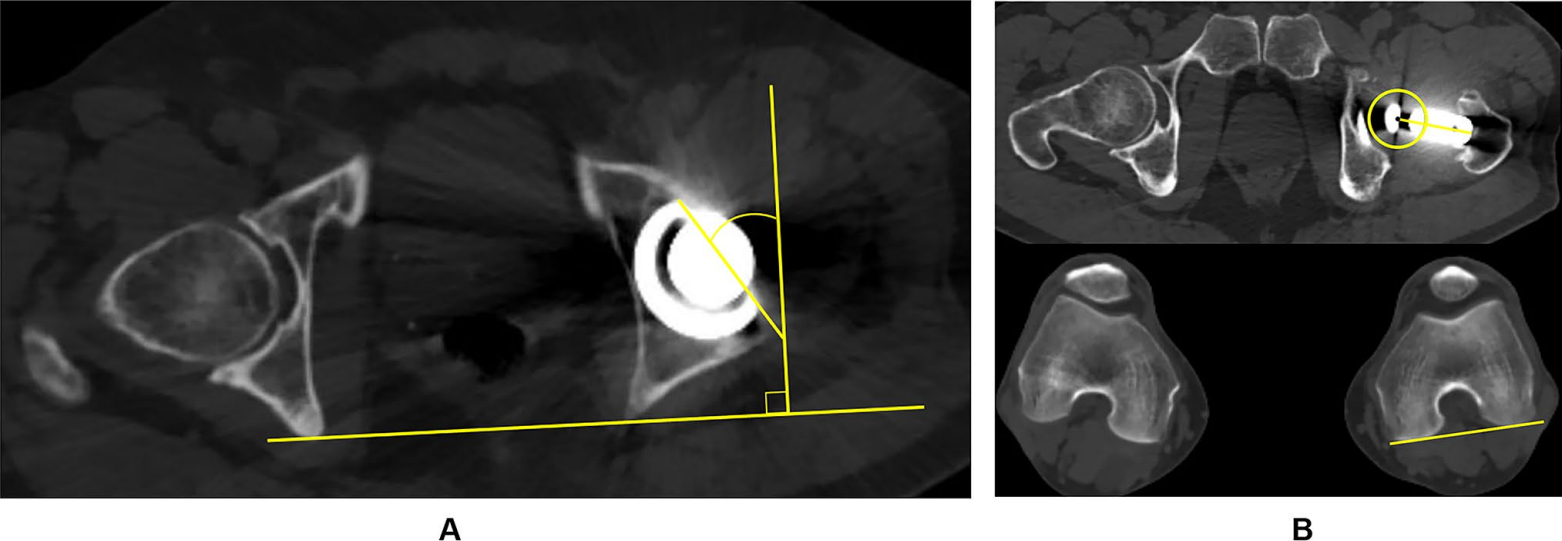
Axial computed tomography (CT) image demonstrating the method used to measure the **A** acetabular anteversion as the line connecting the most anterior and posterior point of the acetabular cup and a reference line drawn perpendicular to a line between the most posterior pelvic margins and **B** femoral anteversion defined as the angle between a line connecting the center of the femoral head to the center of the base of the femoral stem neck and the line connecting the posterior aspect of the medial and lateral femoral condyles

The *CACT* was measured on the axial CT image of the pelvis as the sum of the acetabular cup and femoral stem anteversion. Acetabular cup anteversion was defined as the line connecting the most anterior and posterior point of the acetabular cup at the level of the hip rotational center and a reference line drawn perpendicular to a line between the most posterior pelvic margins (Fig. 3A) [23]. Femoral stem anteversion was defined as the angle between a line connecting the center of the femoral head to the center of the base of the femoral stem's neck and the line connecting the posterior aspect of the medial and lateral femoral condyles (Fig. 3B) [29].

### Computer simulation

**Fig. 4:**
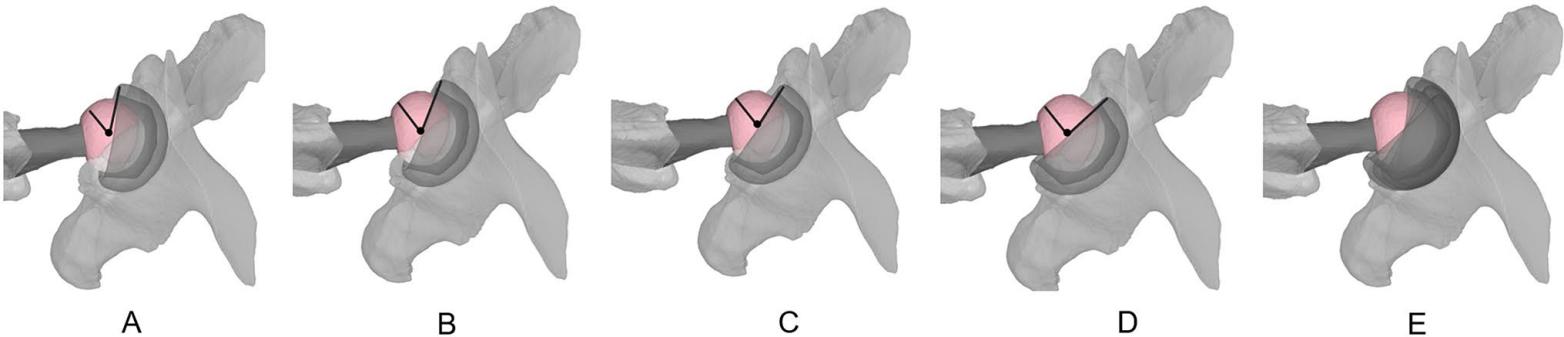
CT-based three-dimensional models of a patient with THA. The acetabular cup anteversion was increased (from 15°, 30°, 45°, 60°) (**A**–**D**) while the cup inclination (40°) femoral stem anteversion (15°) remained constant. Increasing the acetabular cup anteversion (**E**) increased the proportion of the femoral head, which remained uncovered from the acetabular cup

**Fig. 5:**
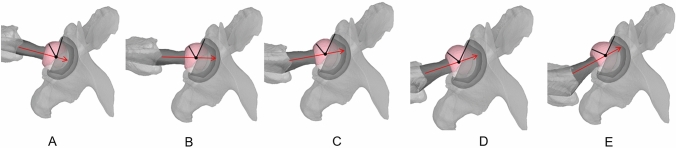
CT-based three-dimensional models of a patient with THA. The femoral stem anteversion was increased (from − 15°, 0°, 15°, 30°, 45°) while the acetabular cup anteversion and inclination remained constant (cup anteversion 30°, cup inclination 40°). Increasing the femoral stem anteversion (from **A**–**E**) increased the portion of the femoral head, which remained uncovered from the acetabular cup. The polar axis (line passing through the center of the stem neck and the center of the prosthetic head) as defined by Pour et al. [25] (red arrow) also moved anteriorly with increased femoral stem anteversion (from **A**–**E**)

As a first step, CT-based three-dimensional models of the entire lower extremity of a patient who underwent a primary THA at our institution were reconstructed. The acetabular cup was implanted at 30° of anteversion and 40° of inclination, whereas the femoral stem at 15° of anteversion. The acetabular cup version was subsequently increased (from 10 to 60°) without changing the inclination and the femoral stem anteversion, with the foot kept in 15° of internal rotation. The view was then set to 45°, to simulate the cross-table lateral hip radiography, and the angle between the femoral head center to the anterior acetabular rim and the base of the femoral head was measured (Fig. 4A–E). Next, the femoral version was increased (from − 15° to 45°) without changing the acetabular version (30°) and inclination (40°) with the foot kept at 15° of internal rotation (Fig. 5A–E). Then the acetabular cup inclination was increased (from 15° to 75°) without changing the acetabular (30°) and femoral (15°) versions with the foot kept at 15° of internal rotation. Finally, the leg was externally rotated from (− 30° to 45°) to investigate without changing the acetabular version (30°), inclination (40°), and femoral stem version (15°) to investigate the effect of leg rotation on the measurement of the radiologic combined anteversion. According to the computer simulation data, the equation of best fit was used to develop a formula that would correct the *CAr* according to the acetabular inclination (*CA_cor*). The *CAr* was then corrected for acetabular cup inclination (*CAr_cor)* measured on an anteroposterior pelvic radiograph to allow for direct comparison with the CA measured on the CT scan (*CACT*).

### Assessment of accuracy and inter-/intra-observer reliability

The accuracy of the *CAr_cor* was defined as the proximity to the *CACT*. The *CAr_cor* was measured by three orthopedic residents (*FC, EW, and CZ*) and one hip fellow *(DD)* blinded to the *CACT*. The average *CAr_cor* of the four above-mentioned measurements was compared to the *CACT*. For the intra-observer reliability, one examiner (*DD*) reassessed the *CAr_cor* after a 4-week interval to avoid recall bias without comparison to the previous measurements. The inter-observer reliability of the *CAr_cor* was assessed by comparing the results of the four examiners. The intra-observer and inter-observer reliabilities of the measurements were evaluated using an average-measure intra-class correlation coefficient (ICC) with a two-way random-effects model for absolute agreement. Plain radiographs were presented to each examiner in random order by a research assistant who did not participate in the reliability and accuracy sessions.

### Subgroup analysis of patients with and without THA dislocation

The *CACT* of patients with and without THA dislocation was compared to detect any differences. The percentage of the THA components with a *CACT* within the “safe zone” of 25–50°, as defined by Dorr [7], was also evaluated.

### Statistical analysis

Descriptive statistics used averages, standard deviation, range, and percentages to present the data. All parameters were tested with the Kolmogorov–Smirnov test for normality. When the criteria for normality were met, a two-tailed *t* test was used. Otherwise, the Wilcoxon signed-rank test was applied. The Bland–Altman plots were used to compare the agreement between the average *CAr_cor* and *CACT* [4]. Pearson correlation coefficient was applied to investigate the relationship between *CAr_cor* and *CACT* but also the effect of cup anteversion, inclination, leg rotation, and femoral stem anteversion of the *CAr.* The level of significance was set to *p* ≤ 0.05. All the statistical analyses were performed using SPSS version 23 software (SPSS Inc., Chicago, Illinois).

## Results

### Radiographic and CT measurements

The average *CAr_cor* was 53 ± 11 (range: 20 to 77)°, whereas the average cup inclination was 39 ± 5 (range: 24 to 55)°. The average acetabular and femoral anteversion, measured on CT, was 34 ± 7 (range: 17 to 59)° and 22 ± 10 (range: -6 to 50)°, respectively, whereas the average *CACT* was 54 ± 11 (range: 21 to 78)°. Patients with THA dislocation demonstrated a significantly higher average *CACT* (62 ± 9)° compared to patients without a THA dislocation (52 ± 10°) (*p* < 0.001). The majority of THAs with and without early dislocation demonstrated a combined anteversion outside the CA” safe zone” (96% and 57%, respectively).

### Computer simulation

**Fig. 6:**
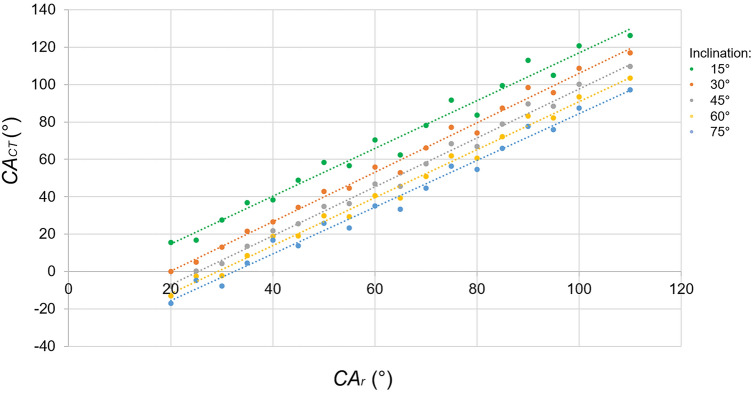
Scatter chart demonstrating the effect of acetabular inclination on the *CAr* measurement. The higher the cup inclination, the higher the *CAr* for the same *CACT.* A linear relationship was found between the *CAr* and *CACT* at all cup inclination angles (*r = *0.99, *p* < 0.001)

The *CAr* was positively correlated to the femoral stem (*r = *0.99, *p* < 0.001) and acetabular cup anteversion (*r = *0.99, *p* < 0.001). The *CAr* was also positively correlated with cup inclination ((*r = *0.99, *p* < 0.001). meaning, that the higher the cup inclination, the higher the *CAr* for the same *CACT* (Fig. 6)*.* The formula to convert the *CAr* to *CAr_cor* was: *CAr_cor = *1.3* *Car *− (17* In (*Cup Inclination*) − 31) (Table 1). The leg rotation was also positively correlated to *CAr* (*r = *0.99, *p* < 0.001), meaning the more externally rotated leg, the more *CAr* would be measured on the lateral cross-table hip radiograph.

**Table 1:** Table of transformation of the radiographic combined anteversion (*CAr*) to corrected combined anteversion (*CAr_cor*) according to acetabular cup inclination

Combined anteversion (°)	Corrected combined anteversion (°)				
	Inclination				
	15°	30°	45°	60°	75°
10	− 4	− 16	− 21	− 24	− 26
20	16	0	− 8	− 13	− 17
25	17	5	0	− 3	− 5
30	28	13	4	− 2	− 8
35	37	21	14	8	4
40	38	26	22	19	17
45	49	34	26	19	14
50	58	43	35	30	26
55	57	45	36	29	23
60	70	56	47	40	35
65	62	53	46	39	33
70	78	66	58	51	45
75	92	77	68	62	56
80	84	74	67	61	55
85	99	87	79	72	66
90	113	99	90	83	78
95	105	96	88	82	76
100	121	109	100	94	87

### Accuracy and inter-/intra-observer reliability

**Fig. 7:**
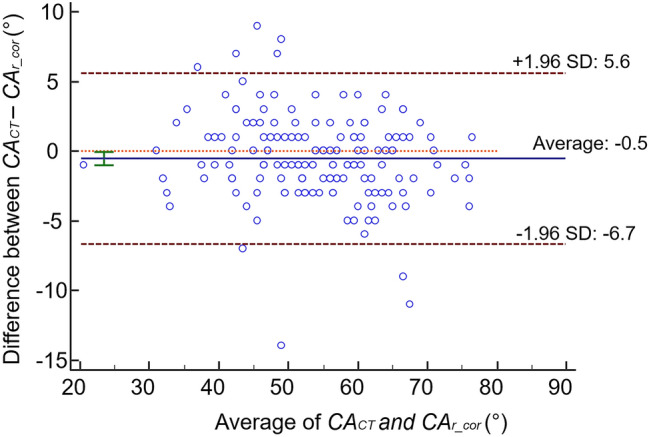
Bland–Altman plot for agreement between *CAr_cor* and *CACT*. *Blue line: bias of *CAr_cor* compared to *CACT*. Green line: 95% confidence interval of the bias. Red dotted lines: 95% limit of agreement between *CAr_cor* and *CACT*. Bias close to zero and narrow limits indicate that the method studied (*CAr_cor*) could replace the gold standard (*CACT*)

Accuracy: The Bland–Altman plot demonstrated an average bias of − 0.5° (95% limits of agreement: − 6.7 to 5.6)° between *CAr_cor* and *CACT* (Fig. 7). Pearson’s correlation demonstrated a strong correlation between *CAr* and *CACT* (*r = *0.96, *p* < 0.001).

Reliability: The intra-observer and inter-observer reliability were in excellent agreement between *CAr* and *CACT* (0.94 and 0.93), respectively.

## Discussion

Adverse clinical outcomes related to implant malposition have stimulated an increasing interest in optimal component orientation in primary THA. Accurate measurement of component anteversion is important in evaluating the radiographic outcomes of THA but also patients with persistent complaints following THA. Although several methods have been proposed for accurate measurement of acetabular cup anteversion on plain radiographs, no data are available on combined component anteversion. The aim of the present study was to evaluate the accuracy and reliability of a novel radiographic method on the lateral hip radiograph in estimating CA in THA. The study demonstrated a high accuracy of the *CAr_cor* with an average bias of − 0.5° compared to the *CACT,* with excellent inter- and intra-observer reliability, suggesting that the *CAr_cor* could be routinely used postoperatively to estimate the CA of the THA components.

The *CAr_cor* essentially represents the proportion of the anterior femoral head, which is not covered by the acetabular cup (Fig. 1). By increasing the acetabular anteversion, it is expected that a larger proportion of the anterior head will be uncovered. A similar effect is anticipated by increasing the femoral stem anteversion. This is confirmed by the computer simulation performed in the present study (Figs. 4 and 5), which demonstrated a positive linear correlation with both femoral stem, and acetabular cup anteversion, meaning that by increasing acetabular or femoral anteversion, the proportion of the femoral head, which remains uncovered from the acetabular cup will also increase. Pour et al. [19] in a simulation study demonstrated that by increasing both acetabular cup and femoral stem anteversion, the polar axis (line passing through the center of the stem neck and the center of the prosthetic head) moved anteriorly, which is in accordance with our observation (Fig. 5).

The importance of CA in establishing an impingement-free range of motion and preventing THA dislocation has been well-described and the suggested “safe zones” are based on simulation studies [25, 27, 28, 30] or clinical observations [21]. However, the surgical approach might affect the CA, and consequently different “safe zones” might be needed for other surgical approaches. Dorr et al. [7] reported an average CA of 37 ± 7° following a navigated THA through a mini-posterior approach in 47 patients, with 96% of the THAs within the defined “safe zone” of 25 to 50°. Li et al. [13] reported an average CA of 23 ± 13° in 501 patients (545), who underwent a THA through the lateral approach, with only 44% of the THAs being within the “safe zone” of 25 to 50°. Nevertheless, no dislocations have been reported. Jackson et al. [9] demonstrated an average CA of 44 ± 10° in 29 patients who underwent a THA through the direct anterior approach, with 79% of the hips being within the “safe zone”. In the present study, the average CA in 148 patients (154 hips) who underwent a THA through the minimally invasive anterior approach was 54 ± 11°, which is markedly higher than the other approaches reported in the literature. The CA in patients without THA dislocation was 52 ± 10°, with 57% of the THAs having a combined anteversion outside Dorr´s “safe zone”. Patients with THA dislocation demonstrated a significantly higher CA (62 ± 8°) with 96% having a CA outside the Dorr´s “safe zone”. These results might suggest that a surgical approach-specific “safe zone” might be necessary to evaluate the risk of THA dislocation.

The present study should be interpreted in light of its potential limitations. The most obvious drawback was that the lateral hip radiograph in the current study was performed in a standardized protocol, by a well-trained staff of our orthopedic department, but as demonstrated in the present study, leg rotation can strongly influence the measurement of CA on the lateral cross-table hip radiograph. Hence, the *CAr_cor* should not be used to measure the CA of THA components in lateral radiographs not performed in the above-mentioned standardized matter, as leg rotation might affect the results. Also, the femoral heads used in the present study had a distinct base, which was clearly identified on lateral radiographs (Fig. 2). Therefore, the current method cannot be used with prosthetic heads which do not have an easily identified base. Furthermore, it should be noted, that only a limited number of stem types (straight tapered and anatomical) were analyzed. Although their design might be similar to other implants available, other stems should be evaluated to confirm the findings of the current study.

In conclusion, the result of the present study suggests that the *CAr_cor* measurement on cross-table lateral hip radiographs is accurate and reliable, implying that it could be routinely used postoperatively to estimate the CA of the THA components. More patients should be recruited in future studies to confirm the validity and accuracy of the *CAr* on a larger scale and with different THA designs.

## Data Availability

The materials described in the manuscript, including all relevant raw data, will be freely available to any researcher wishing to use them for non-commercial purposes, without breaching participant confidentiality.
